# The Preliminary Training School for Nurses: I. Its Uses and Developments

**Published:** 1917-05-26

**Authors:** 


### THE MATRONS' AND SISTERS' DEPARTMENT.

### THE PRELIMINARY TRAINING SCHOOL FOR NURSES.

### I. Its Uses and Developments.

IN these days when there is a greater need than ever before of trained women, it is necessary to look into our present systems of training, and see in what directions we can improve. In every kind of work so much depends on the start, and in practically every work that counts there is some kind of preparation before the actual work can be intelligently undertaken. Though, of course, sick nursing must of necessity be taught by actual attendance on the sick, there is very much that can be learnt in a few weeks in a Home, under trained supervision, before beginning work actually in the hospital wards, where the training of probationers must necessarily come second to the care of the patients. In busy wards it is extremely difficult to give much time to the actual and clear teaching of preliminaries, and much has to be " picked up " by quite untrained women, who, though they may be intelligent and anxious to learn, must of necessity be ignorant of the essential points to be attended to, and so they rush through the work, simply aiming, in many cases, at getting it finished.

One advantage of a Preliminary Training School is that probationers are taught to live with others in community life, and have the opportunity of acquiring habits of method, concentration, punctuality, and thoroughness before they start the entirely new life they will lead in hospital. Those who have never left home before do not feel so lost and lonely when they start with twenty or thirty as new as themselves, so they get a better chance of adapting themselves mentally and physically to changed conditions than they would do if they entered the hospital in the first instance.

As sick nursing is a profession that is taken up by women of almost every social grade, it is all the more desirable that the routine duties should be taught under skilled supervision, before the actual nursing is attempted. These could include sweeping without knocking the legs of beds or making a noise, dusting thoroughly so that high ledges, etc., are kept clean, making beds, both with and without a dummy patient; lifting, turning, and supporting patients, and the most convenient methods of washing and attending to them; reading the clinical thermometer quickly and accurately, taking the temperature, pulse, etc.; learning splint padding, bandaging, and invalid cookery.

In addition to this, an elementary knowledge of hygiene and physiology can be acquired, so that a more intelligent interest is taken in the diseases and injuries of the patients on entering hospital, as many who wish to become nurses do not even know the names and positions of the different organs of the body. While the better educated women will find the theoretical work, which must be elementary, quite easy and the manual work more difficult, those who have had a more limited education, but a domestic training, will find their difficulties lie in the opposite direction, so this makes it quite possible to train both under the same curriculum.

It is quite clear that the sister who is in charge of the training school would have a much better chance of judging the fitness of candidates for training after having each one individually under careful Home observation for a few weeks. This has the twofold merit of eliminating candidates who are obviously unfitted for the work before they have given any trouble in hospital. If those in charge are good judges of character, they can prevent undesirables slipping through and getting a certificate, or, at any rate, a long term of training of which the hospital must eventually bear the loss. Inefficiency and unreliability are much easier to hide when there are large numbers, and when the sisters are occupied with the patients as well as with the nurses, so the bad worker is not so easily detected. Also, the preliminary training ensures that all who are placed under ward sisters with a view to regular training are able at once to take an intelligent share in the routine work of the ward.

It is much easier for a sister to judge whether a candidate, who at first seems dull, is really incapable or only shy and self-conscious; also by living with the probationers she is much better able to judge of physical, mental, and temperamental suitability. A candidate might have plenty of latent capability which careful Home training would develop. But, if overwhelmed by the feeling that things must be " got through," and that she is in the way by not understanding in the least what is wanted, and by lack of knowledge of even the names of things required, those who might make good nurses if they had a reasonable start may become disheartened, and so be lost to the Nursing Profession.

In a Preliminary Training School, those who have only taken up nursing as a " fad " will soon give up of their own accord when they find that nursing means hard work and self-denial; for to be of real use this Home must be run on definite hospital lines, only without patients, a life-size dummy serving as a substitute.

As hospitals are built and supported primarily for the purpose of looking after those sick people who are too poor to pay for proper attention in their own homes, it scarcely seems just, if it can be avoided, that the whole training should be in the wards, and that sick people should run the risk of having some of their wants attended to by quite unskilled hands. So many well-meaning people " take up " nursing, without even thinking what harm they may do through ignorance. Enough can be taught to beginners beforehand, in a few weeks, by trained people who give their whole time and attention to the matter, to prevent unnecessary discomfort to the patient, and enable a new probationer to realise the importance of " listening " carefully to an order, and obeying it.

